# Supplementary figures and images for: Spatial and Temporal Pattern of Net Ecosystem Productivity in China and Its Response to Climate Change in the Past 40 Years
Source: Int J Environ Res Public Health. 2022 Dec 21;20(1):92. doi: 10.3390/ijerph20010092 (PMC9819965; doi:10.3390/ijerph20010092)

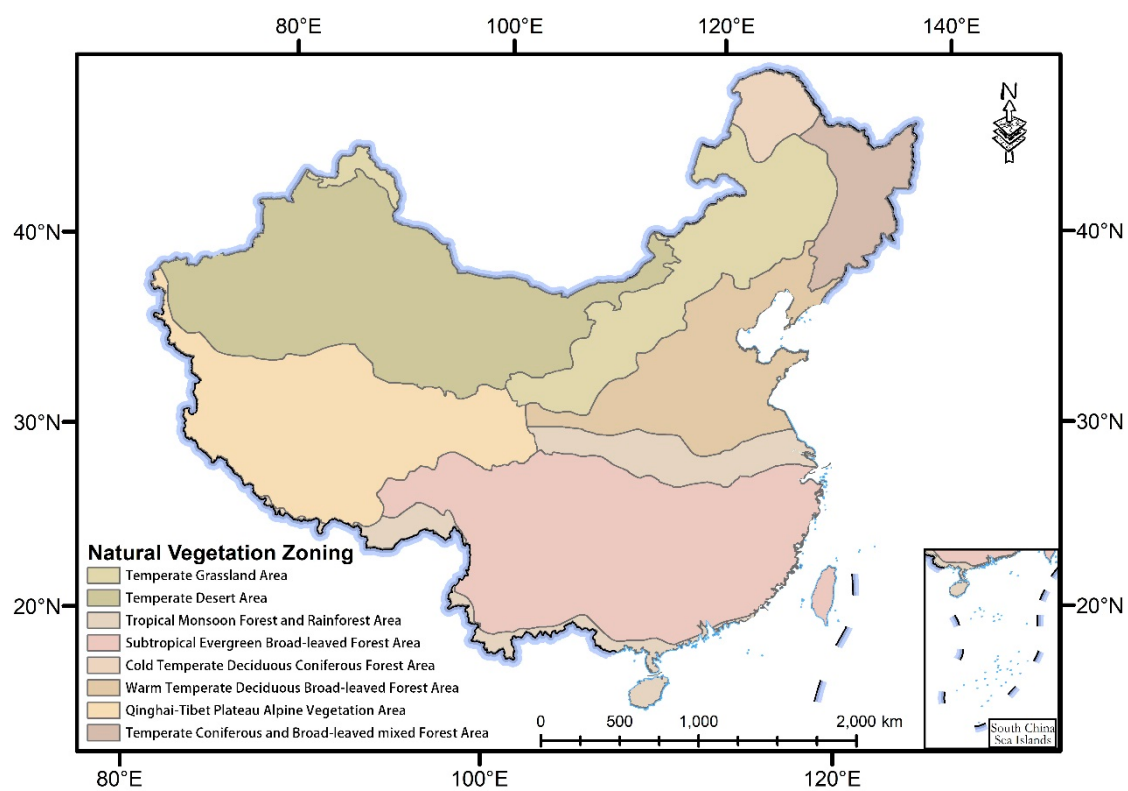

**Figure S1** Natural Vegetation Zoning of China.

Supplement: Supplementary file 1 [file ijerph-20-00092-s001.zip › ijerph-2053529-supplementary.pdf]
